# EscaYard: Precision viticulture multimodal dataset of vineyards affected by Esca disease consisting of geotagged smartphone images, phytosanitary status, UAV 3D point clouds and Orthomosaics

**DOI:** 10.1016/j.dib.2024.110497

**Published:** 2024-05-03

**Authors:** Sergio Vélez, Mar Ariza-Sentís, João Valente

**Affiliations:** Information Technology Group, Wageningen University & Research, 6708 PB Wageningen, the Netherlands

**Keywords:** Viticulture, Precision Agriculture, Disease, Drone, RTK GNSS Data, Woody crop

## Abstract

The “EscaYard” dataset comprises multimodal data collected from vineyards to support agricultural research, specifically focusing on vine health and productivity. Data collection involved two primary methods: (1) unmanned aerial vehicle (UAV) for capturing multispectral images and 3D point clouds, and (2) smartphones for detailed ground-level photography. The UAV used was DJI Matrice 210 V2 RTK, equipped with a Micasense Altum sensor, flying at 30 m above ground level to ensure detailed coverage. Ground-level data were collected using smartphones (iPhone X and Xiaomi Poco X3 Pro), which provided high-resolution images of individual plants. These images were geotagged, enabling location mapping, and included data on the phytosanitary status and number of grape clusters per plant. Additionally, the dataset contains RTK GNSS data, offering high-precision location information for each vine, enhancing the dataset's value for spatial analysis. Moreover, the dataset is structured to support various research applications, including agronomy, remote sensing, and machine learning. It is particularly suited for studying disease detection, yield estimation, and vineyard management strategies. The high-resolution and multispectral nature of the data allows for a detailed analysis of vineyard conditions. Potential reuse of the dataset spans multiple disciplines, enabling studies on environmental monitoring, geographic information systems (GIS), and precision agriculture. Its comprehensive nature makes it a valuable resource for developing and testing algorithms for disease classification, yield prediction, and plant phenotyping. For instance, the images of bunches and grape leaves can be used to train object detection algorithms for accurate disease detection and consequent precise spraying. Moreover, yield prediction algorithms can be trained by extracting the phenotypic traits of the grape bunches. The “EscaYard” dataset provides a foundation for advancing research in sustainable farming practices, optimising crop health, and improving productivity through precise agricultural technologies.

Specifications Table

**Table:** 

Subject	Agricultural Sciences, Agronomy and Crop Science
Specific subject area	Precision Agriculture for disease detection and yield estimation using remote and proximal sensing using UAVs and smartphones
Type of data	“HEIC” and “JPG”: Geotagged smartphone images “.csv” datasheet: description of the phytosanitary and yield data “.tif”: UAV orthomosaics “.las”: 3D point clouds “Shapefile” format: RTK GNSS data with the precise plant locations.
Data collection	Data were collected using UAV DJI Matrice 210 V2 RTK for aerial multispectral imagery and smartphones (iPhone X, Xiaomi Poco X3 Pro) for ground-level photos, both documenting the number of grape clusters and the health of each plant. UAV flights, set at 30m AGL, captured imagery across six spectral bands. Smartphone images, geotagged for precise location, complemented UAV data for a detailed analysis. Data processing involved PIX4D for 3D point clouds and orthomosaics. The plants were accurately georeferenced using a Trimble R2 GNSS system.
Data source location	Institution: Wageningen University & Research, The Netherlands City/Town/Region: Tomiño, Pontevedra, Galicia Country: Spain Coordinates: Vineyard B7, X: 517183.8, Y: 4645072.8; Vineyard B9, X: 516987.8, Y: 4644823.7 (ETRS89 / UTM zone 29N, EPSG:25829).
Data accessibility	Repository name: Zenodo Data identification number: https://zenodo.org/doi/10.5281/zenodo.10362567 Direct URL to data: https://zenodo.org/records/10362567

## 1 Value of the Data

• The dataset offers a unique combination of multimodal data, including geotagged smartphone images, UAV orthomosaics, 3D point clouds, and precise geolocation data, enabling a multifaceted analysis of vineyard health and productivity.
• RTK GNSS data facilitates high-precision monitoring and management of vineyards, and with accurate geolocation data, researchers can perform geospatial analyses to understand the distribution of diseases, yield variability, and other factors at a micro-scale within the vineyard.
• The dataset allows yield estimation, critical for planning and economic forecasting in viticulture, and enables early detection and monitoring of diseases such as Esca, critical for crop health, thus supporting sustainable farming practices.
• The diverse data types, including multimodal imagery and structured data, make the dataset an excellent resource to support Machine Learning applications and for developing and testing Deep Learning models for tasks like disease classification, yield prediction, and phenotyping.
• The dataset promotes cross-disciplinary studies in viticulture, remote sensing, agronomy, and data science, enhancing innovation and collaboration. It enables new methods for plant health, phenotyping, and precision agriculture, advancing research techniques.
• The possibility of combining this dataset with other datasets gathered from the same vineyard (such as [[1], [2], [3]], which contain multispectral images, RGB videos and LiDAR data), enhances its value, enabling comprehensive studies on vineyard ecosystems, disease dynamics, and the impact of agricultural practices on crop health and the environment.
• Increased flexibility and convenience for students conducting preliminary research, as they can efficiently utilize smartphones for survey purposes, streamlining their data collection efforts.

## 2 Background

Agricultural diseases significantly impact global food production, sustainability, and profitability. Diseases such as *Botrytis cinerea* in grapes [4,5], *Phytophthora infestans* in potatoes and tomatoes [6], and head blight in wheat and barley caused by *Fusarium* spp [7] lead to crop losses and lower harvest quality. These pathogens threaten food security and have economic consequences, necessitating early detection methods. Specifically in viticulture, Esca disease is a major threat in viticulture, causing grapevine decline and death with symptoms like “tiger stripes” on leaves and wood necrosis, significantly affecting yield and wine quality [8]. Early detection and handling of Esca disease are crucial due to its complexity and late symptom onset. On the other hand, effective yield management is crucial for food security, economic stability, and environmental health. It plays a key role in meeting global food demand, reducing hunger and poverty, especially in agriculture-dependent developing countries [9].

Precision agriculture can help in these tasks, enhancing crop management, productivity, and resource conservation by employing technologies such as high-precision positioning systems, drones or unmanned aerial vehicles (UAVs), and artificial intelligence (AI). These technologies allow for the systematic collection and analysis of data, which can then be processed using algorithms, including Machine and Deep Learning for disease control, phenotyping and yield management [[10], [11], [12]]. In this context, the contribution of comprehensive datasets, like the one detailed in this research, is essential.

## 3 Data Description

This study presents “EscaYard”, a dataset gathered from vineyards, which comprises multimodal data from vineyards, consisting of 253 geotagged smartphone ground images, one datasheet describing the phytosanitary status and number of grape clusters, ten 3D point clouds, two UAV orthomosaics, and RTK GNSS data with trunk locations, providing a multi-faceted view of the vineyards through the combination of high-resolution ground truth with aerial perspectives and accurate geolocations. Table 1 shows a general overview of the dataset, including the name of the files and their size. It is composed of geotagged smartphone ground images comprised in “.7z” format, a “.csv” file including the phytosanitary status of each plant, UAV 3D Point Clouds in “.las” format, UAV Orthomosaics in “.tif” format, and RTK GNSS Data of the location of the plants in “.shp” format.

**Table 1:** File names and size of each file.

File name	File size
20220714_FLEXIGROBOTS_B7_30M0G_MS_PRO_NIR_densified_point_cloud.las	235.54 MB
20220714_FLEXIGROBOTS_B9_30M0G_MS_PRO_Red_densified_point_cloud.las	124.82 MB
20220714_FLEXIGROBOTS_B7_30M0G_MS_PRO_Blue_densified_point_cloud.las	216.52 MB
20220714_FLEXIGROBOTS_B7_30M0G_MS_PRO_Red_densified_point_cloud.las	220.31 MB
20220714_FLEXIGROBOTS_B9_30M0G_MS_PRO_Red edge_densified_point_cloud.las	133.98 MB
20220714_FLEXIGROBOTS_B9_30M0G_MS_PRO_Blue_densified_point_cloud.las	127.35 MB
20220714_FLEXIGROBOTS_B7_30M0G_MS_PRO_Red edge_densified_point_cloud.las	232.81 MB
20220714_FLEXIGROBOTS_B7_30M0G_MS_PRO_Green_densified_point_cloud.las	223.85 MB
20220714_FLEXIGROBOTS_B9_30M0G_MS_PRO_NIR_densified_point_cloud.las	134.29 MB
20220714_FLEXIGROBOTS_B9_30M0G_MS_PRO_Green_densified_point_cloud.las	131.42 MB
PLANTs_SmartPhonePhotos_HEIC.7z	452.90 MB
PLANTs_SmartPhonePhotos_JPG.7z	1.49 GB
20220714_FLEXIGROBOTS_B7_30M0G_MS_PRO.tif	3.37 GB
20220714_FLEXIGROBOTS_B9_30M0G_MS_PRO.tif	1.52 GB
trunk_locations_VineyardB7.zip	8.45 KB
datasheet.csv	27.61 KB

### 3.1 Smartphone images

**Fig. 1:**
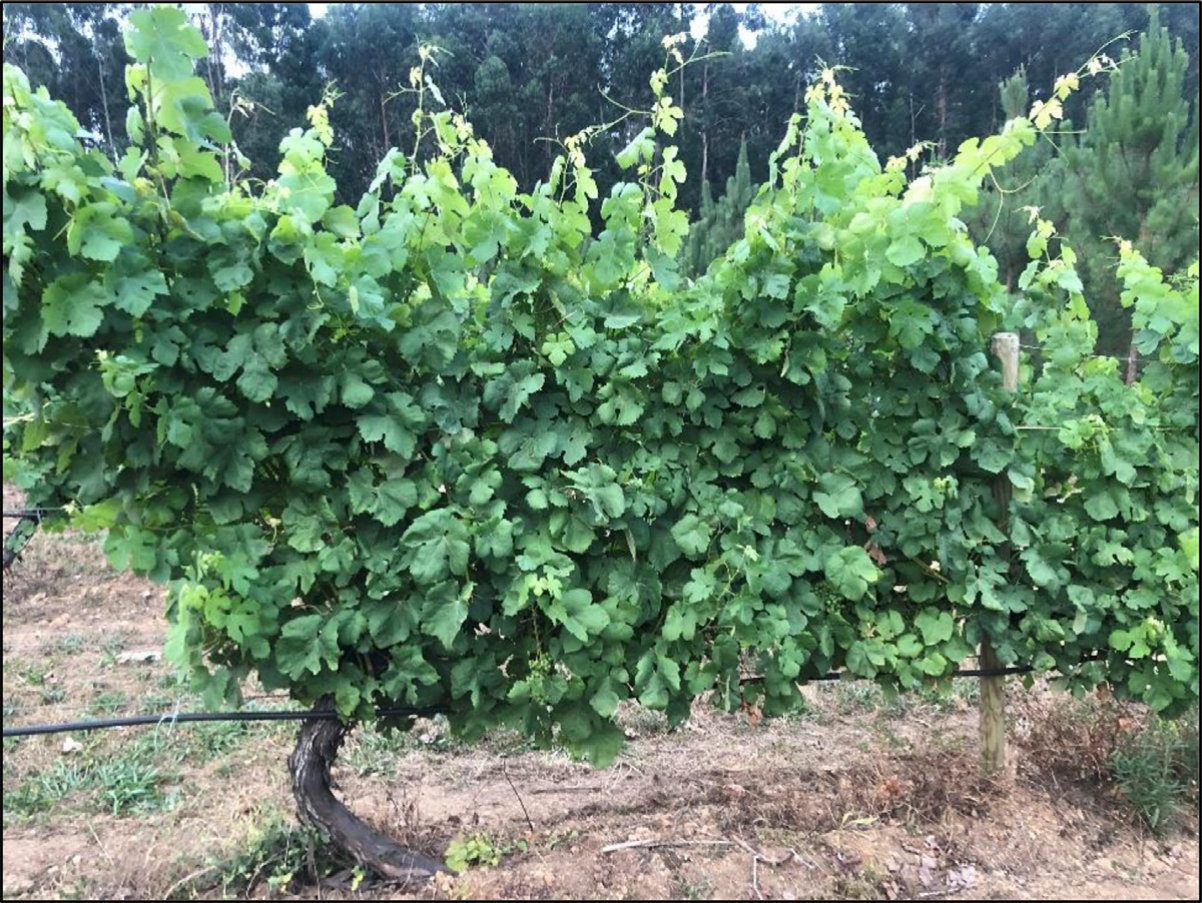
Example of an image taken with the smartphone.

The archive files “PLANTs_SmartPhonePhotos_HEIC.7z” and “PLANTs_SmartPhonePhotos_JPG.7z” pack together 253 full plant snapshots (Fig. 1), captured using smartphones. Android devices primarily utilize the JPG format, whereas Apple devices opt for HEIC. JPG, a well-known format in digital imagery, employs a lossy compression technique which might degrade quality at higher compression levels. It delivers an 8-bit colour depth per channel without support for transparency, yet its broad compatibility ensures its ease of use across numerous devices and applications. Conversely, the HEIC format supports a more advanced and efficient compression technology, achieving smaller file sizes without quality compromise. HEIC stands out with a 10-bit colour depth per channel, offering more vibrant and detailed visuals along with transparency support, which is an advantage for graphic design. However, its compatibility is lower than JPG, often necessitating conversion for non-Apple device usage platforms [13]. Therefore, to aid in broader research applications, HEIC images have also been converted to JPG. Furthermore, the image metadata includes the GPS coordinates of the location of each photo, taken with the mobile phone, alongside other valuable data such as the model of the capturing device and the timestamp of the photo.

Concerning the naming convention for files on an Android phone, it is structured as follows: “IMG_YYYMMDD_HHMMSS.jpg”, where “IMG” signifies “Image,” “Y” represents the “year,” “M” denotes the “month,” and “D” indicates the “day.” Moreover, “H” stands for the “hour,” “M” for the “minute,” and “S” for the “second” at which the image was captured. For instance, “IMG_20220714_212141.jpg” would be the filename in JPG format for an image captured on July 14, 2022, at 21 h, 21 min, and 41 s, that is, at 21:21:41.

Similarly, the naming scheme for images from an Apple phone is “IMG_XXXX.HEIC”, where “IMG” translates to “Image,” and “X” corresponds to the consecutive image number. As an example, “IMG_9423.HEIC” would refer to image number 9423. In a similar vein, iPhone images that are converted to JPG will follow the pattern “IMG_XXXX.JPG”.

### 3.2 Phytosanitary status and yield

The “.csv” file contains information about the position of the plants (the geolocation of their trunk) in WKT format and contains information about the health status of plants concerning the disease complex of Esca. Note that this disease stands as the most widespread affliction within the vineyard at that time, thereby capturing the primary attention of the technicians of the winery for identification purposes. However, it is important to acknowledge that other diseases may also be present, albeit at a significantly lower incidence rate. Each row in the “.csv” corresponds to a specific grapevine and contains the following information:

- WKT (Well-Known Text): This column holds the spatial reference in POINT format, providing the precise longitude and latitude of each grapevine.
- File: The name of the image file associated with each data point, representing a geotagged photo of the grapevine taken with a smartphone.
- Number of Grape Clusters: This column records the count of grape clusters for each plant counted in the field. All bunches, including those visible to the naked eye and those hidden under the leaves, were counted from 42 plants in the vineyard.
- Esca: This is a binary indicator, marked “YES” or “NO”, indicating whether the grapevine is affected by Esca disease. The determination is made by an expert agronomist who evaluates the symptoms on the leaves, specifically looking for “tiger stripes” patterns.
- Time: The time of day when the image was taken, which can be listed as “midday” or “evening”.
- Vineyard: The specific vineyard where the grapevine is located.
- Mobile Device: The type of smartphone used to take the image.
- X, Y, Z: These columns represent the coordinates of the grapevines in the coordinate reference system ETRS89 / UTM zone 29N, EPSG:25829.

### 3.3 UAV orthomosaics

**Fig. 2:**
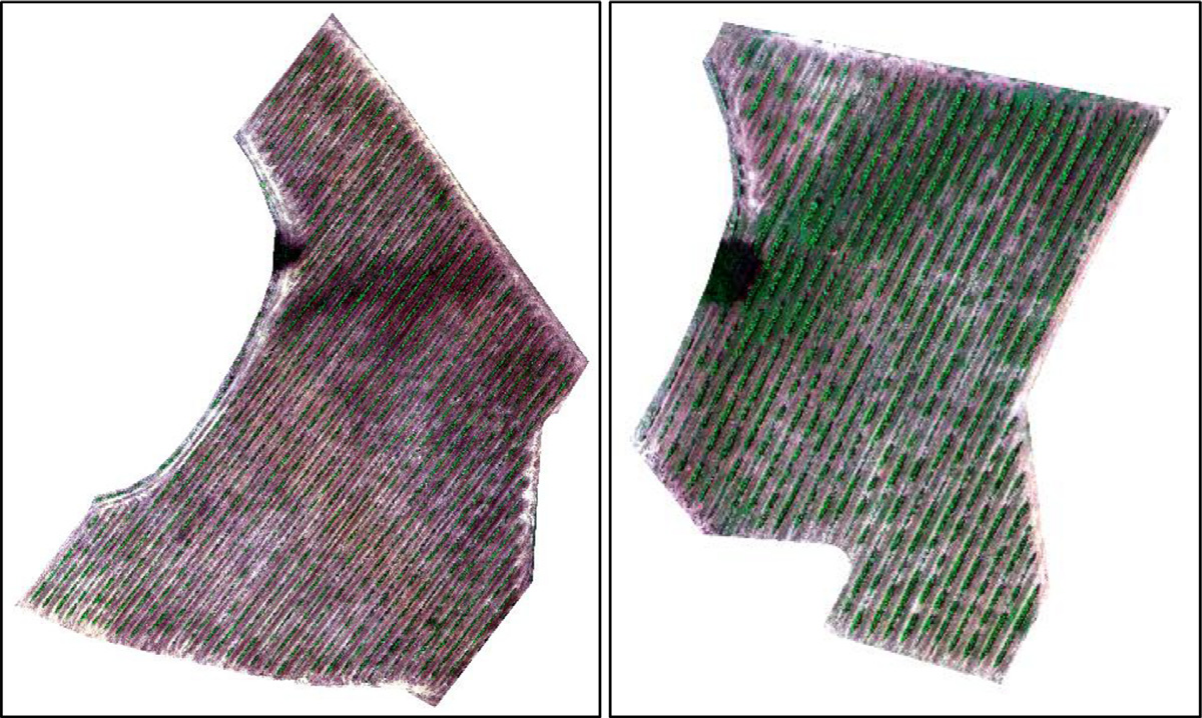
RGB orthomosaics depicting the vineyards. Left: vineyard B7. Right: vineyard B9.

This dataset includes two orthomosaics covering the entire vineyards (Fig. 2). The coordinate reference system is ETRS89 / UTM zone 29N, EPSG: 25829. Their spectral composition includes five bands: blue (1), green (2), red (3), red edge (4), and near-infrared, NIR (5), allowing the generation of vegetation indices such as NDVI, useful for assessing vineyard vegetation status and yield potential [14,15]. The file naming protocol adopted for this collection is as follows: “YYYYMMDD_FLEXIGROBOTS_BX_XXMXG_MS_PRO.tif”, where “YYYYMMDD” is the capture date of the images, with “YYYY” for the year, “MM” for the month, and “DD” for the day. “FLEXIGROBOTS” provides the project identifier. “BX” denotes the specific vineyard from which the images were taken, “XXM” refers to the flight altitude, and “XG” details the tilt angle of the camera, relative to the nadir. Moreover, “MS” indicates the orthomosaic is multispectral, “PRO” that the imagery has undergone high-quality processing, and the “.tif” extension specifies that the file format is TIF.

For instance, in a file labelled “20220714_FLEXIGROBOTS_B7_30M0G_MS_PRO.tif”, “20220714” would indicate the orthomosaic was generated from images captured on July 14, 2022, “FLEXIGROBOTS” identifies the project, “B7” points to the particular vineyard of interest, “30M” specifies the flight height, here 30 m, and “0G” describes the positioning angle of the camera compared to the nadir. Additionally, “MS” underscores that the orthomosaic is multispectral, “PRO” indicates the high-quality level of image processing applied, and “.tif” specifies the TIF format of the file.

### 3.4 UAV 3D point clouds

**Fig. 3:**
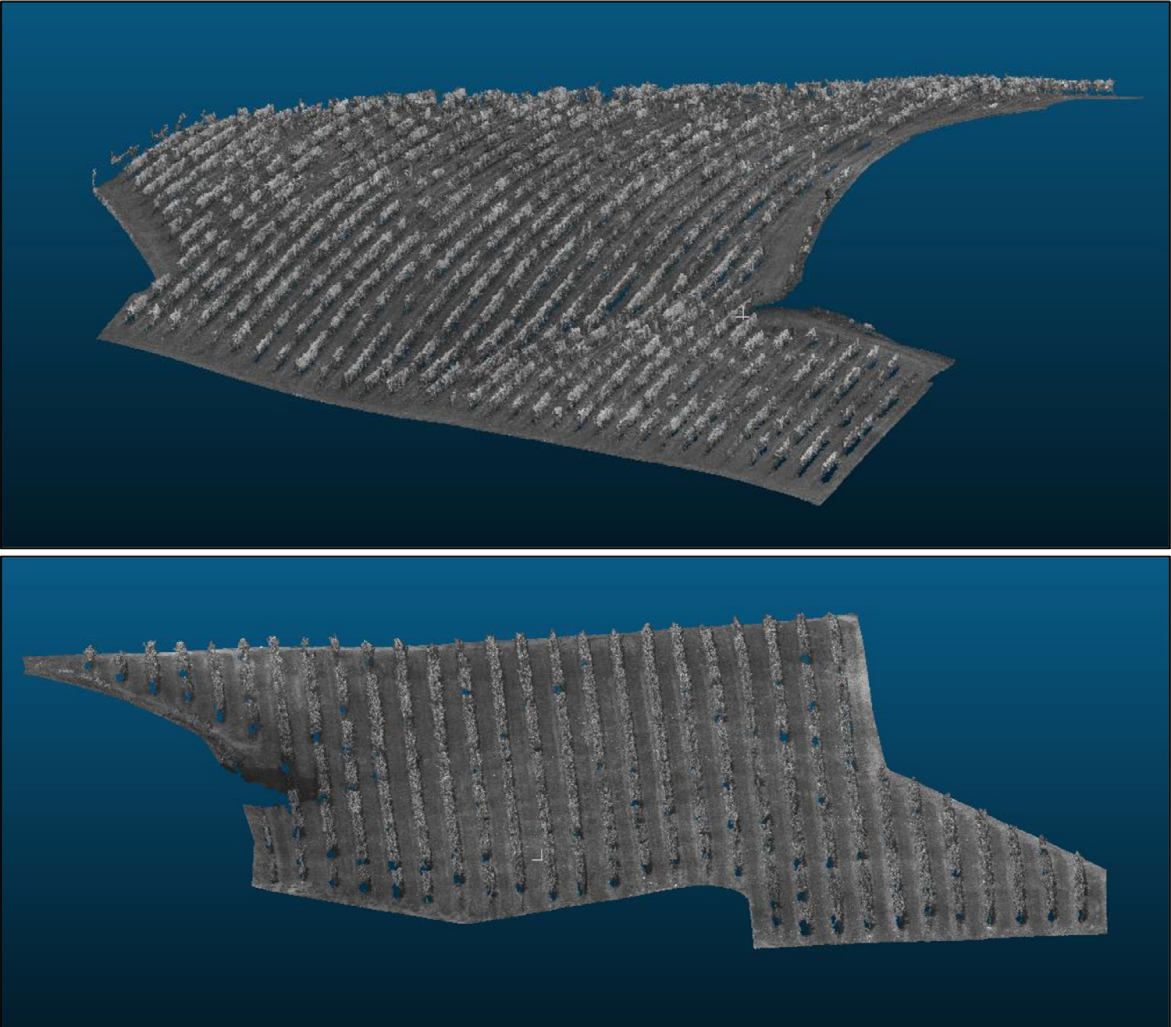
Point clouds of the vineyards generated using Structure from Motion. Green channel. Top: vineyard B7. Bottom: vineyard B9.

This dataset contains ten 3D point clouds, derived from multispectral imagery via the structure from motion (SfM) algorithm (Fig. 3). In contrast to the orthomosaics, these point clouds are supplied with a singular band each, aiming to diminish file size and enhance usability. The naming convention for 3D point clouds in this dataset is structured as follows: “YYYYMMDD_FLEXIGROBOTS_BX_XXMXG_MS_PRO_BAND_densified_point_cloud.las”, where the first portion, “YYYYMMDD_FLEXIGROBOTS_BX_XXMXG_MS_PRO”, follows the same format as in the orthomosaics and, in addition, “BAND” indicates the spectral band, “densified_point_cloud” indicated the point cloud has been densified to enhance the spatial resolution and finally “.las” specifies the file format as “.las”, which is a standard format for LiDAR data and supports the storage of three-dimensional point cloud data.

For instance, a file named “20220714_FLEXIGROBOTS_B7_30M0G_MS_PRO_Green_densified_point_cloud.las” would indicate that the images were collected on July 14, 2022, as part of the FLEXIGROBOTS project, from vineyard B7, at a flight altitude denoted of 30 m, with a specific camera nadir angle. The “MS” suggests the images were multispectral, “PRO” indicates professional processing, and “Green” indicates the specific spectral band, in this case, green. Everything is compiled into a densified point cloud format in a “.las” file.

### 3.5 Locations of the trunks

The file “trunk_locations_VineyardB7.zip” contains the locations of each plant in Shapefile format. This format was created by Esri (Environmental Systems Research Institute), and it is commonly used in Geographic Information Systems (GIS) software. The Shapefile is composed of a set of files with the following extensions:

- “.shp”: contains the spatial geometries of the geographic entities, in this case, points representing the locations of the trunks of the vines in the vineyard B7.
- “.shx”: index file of the geometric entities that allows programs to read the geometry of the .shp file efficiently.
- “.cpg”: specifies the character encoding used to correctly interpret the text data in the Shapefile
- “.dbf”: contains the attribute information in a standard database format, with each database row linked to a geographical entity in the .shp file.
- “.prj”: saves the map projection data and specifies the coordinate reference system, in this case, ETRS89 / UTM zone 29N, EPSG:25829.
- “.qix”: spatial index file that facilitates fast searches and query performance within spatial data.

The locations of the plants in vineyard B9 in Shapefile format can be found published in a previously published dataset [1] and, alternatively, the coordinates can also be retrieved from the “datasheet.csv” file.

### 3.6 Practical application of the dataset: tracking vineyard disease

**Fig. 4:**
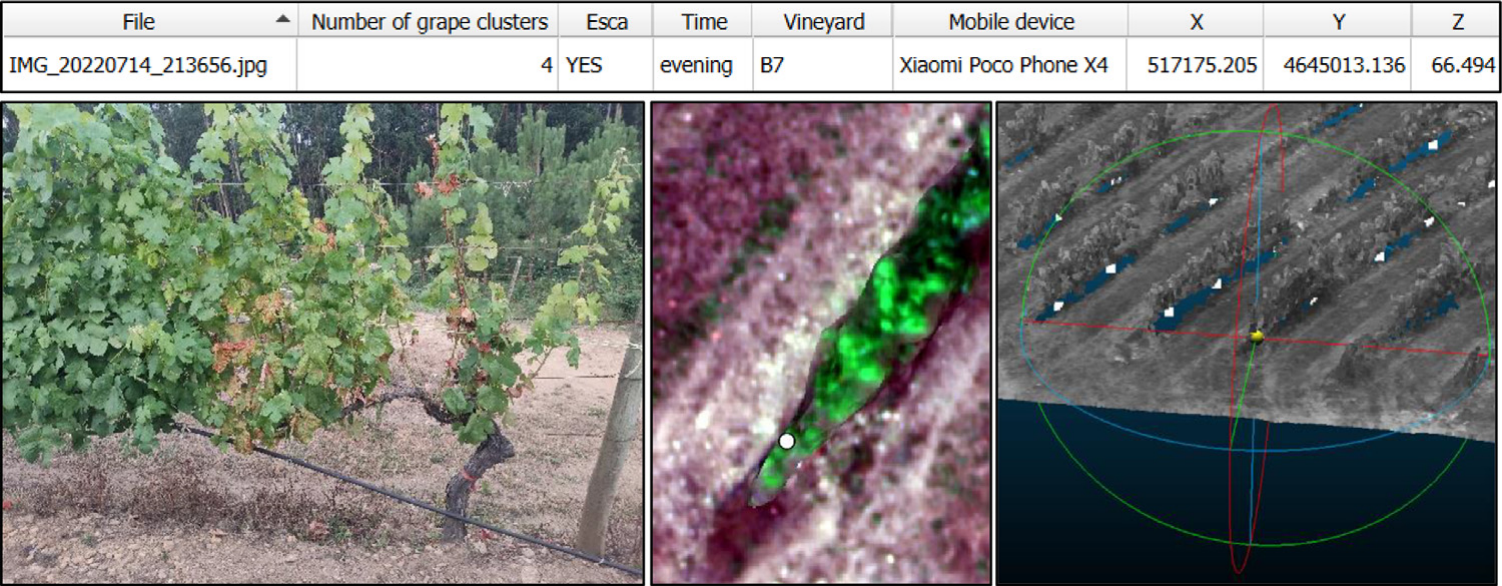
Muti modal data representing the same plant affected by Esca disease. Top: attribute table of the “.csv” indicating detailed information about the plant. Bottom-left: photograph taken on the ground with the mobile device specified in the table. Bottom-middle: detail of the location of the plant (white point) in the multispectral orthomosaic. Bottom-right: 3D point cloud representing the vineyard and plant locations (white points), centred on the studied plant.

The main potential of this dataset is based on the multimodal data it contains. The “EscaYard” dataset enables the detection and monitoring of disease spread, essential for the accurate assessment of affected areas. In this way, Fig. 4 shows an example of an application showing multimodal data from the same plant that are related to each other, providing an integrated view that combines terrain imagery, spectral and geospatial data, along with tabulated information for the documentation and analysis of vine health in a field study.

At the top is displayed the record extracted from the “.csv” file opened in QGIS software (version 3.28.X, QGIS developer team 2023) for the grapevine belonging to the image “IMG_20220714_213656.jpg”. This information includes:

- “Number of grape clusters”: 4, which is the actual count of clusters counted in the field.
- “Esca”: “YES” indicates that the vine shown has signs of Esca disease.
- “Time”: “evening” indicates that the photo was taken in the evening.
- “Vineyard”: “B7” identifies the vineyard where the image was taken.
- “Mobile device”: Xiaomi Poco Phone X4 is the model of the smartphone used to take the photo.
- “Coordinates X, Y, Z”: These are the geospatial coordinates that correspond to the location of the vine in the vineyard, allowing for its precise identification and tracking.

In the lower left, there is the photo captured in the vineyard using the smartphone listed in the table, where the impact of Esca disease can be observed. The image at the centre bottom reveals the location of the plant as recorded by the Trimble R2 device (marked by a white dot) against the background of the multispectral orthomosaic. The image on the lower right shows the 3D point cloud centred on the plant under study, where white dots highlight the locations of plants mapped using the Trimble R2 instrument.

In addition, this dataset can be combined with other datasets on the same disease [16,17], or with existing datasets from the same vineyard [[1], [2], [3]] to enhance the understanding of vineyard ecosystems. By integrating these datasets, researchers can conduct a more detailed analysis of grape bunch visibility, phenotypic traits, disease assessment, and yield estimation under different conditions. This integration could lead to more effective strategies in both Precision Agriculture and Precision Viticulture, offering insights into crop health, yield, and growth patterns. Such an approach highlights the evolving nature of agricultural technologies, where data integration plays a key role in advancing farming practices and vineyard management.

## 4 Experimental Design, Materials and Methods

**Fig. 5:**
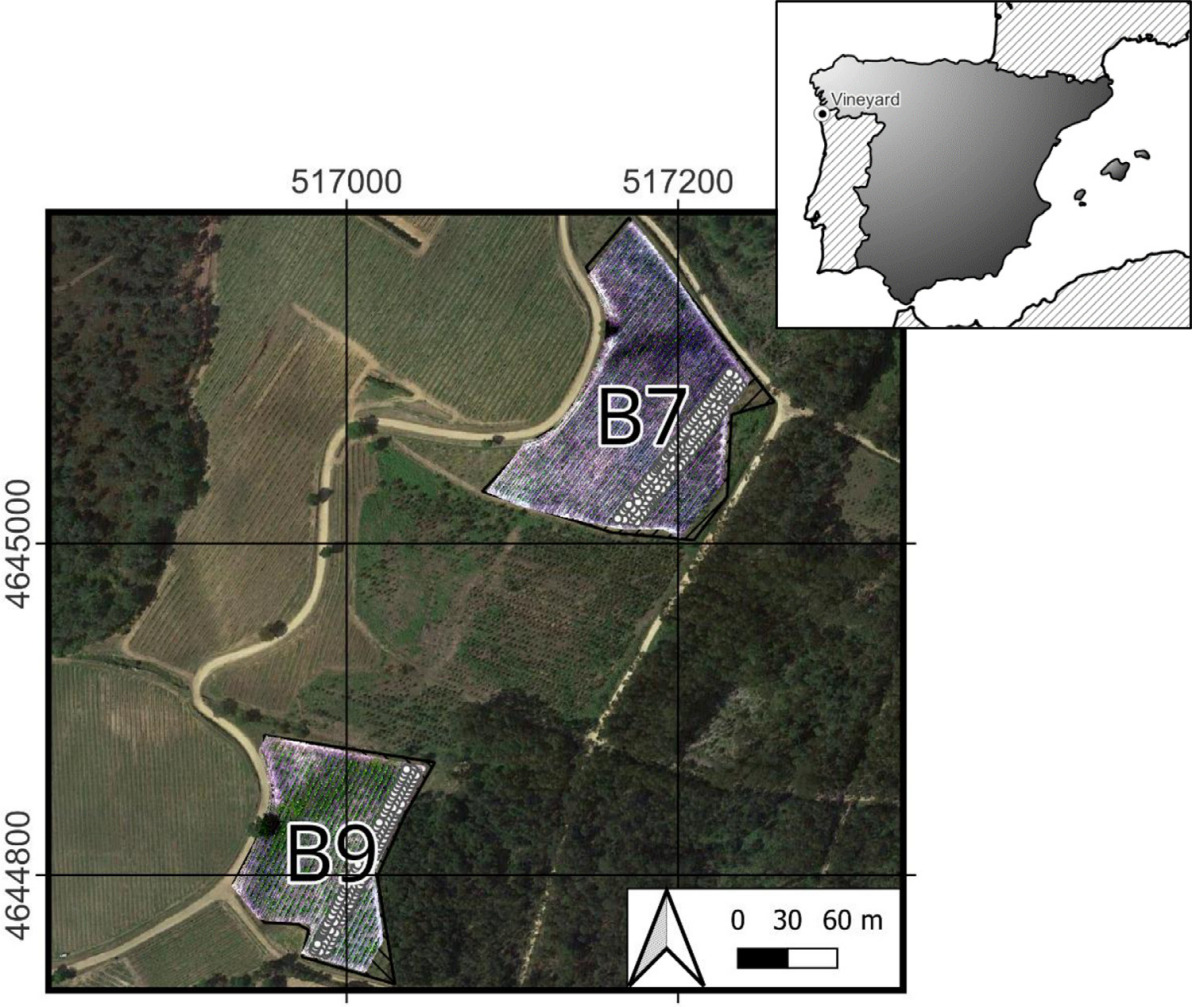
Location of the plants and the vineyards, in Tomiño, Pontevedra, Spain. Coordinates: Vineyard B7, X: 517183.8, Y: 4645072.8; Vineyard B9, X: 516987.8, Y: 4644823.7 (ETRS89 / UTM zone 29N, EPSG:25829).

The study focused on two commercial vineyards of the Vitis vinifera cv. Loureiro variety, identified as B7 and B9 (Fig. 5). These vineyards are owned by “Bodegas Terras Gauda S.A.” and are situated in Tomiño, Pontevedra, in the Galicia region of Spain (Coordinates in WGS 84 / UTM zone 29N, EPSG:32629: Vineyard B7, X: 517186.7, Y: 4645072.3; Vineyard B9, X: 516987.9, Y: 4644817.7). The vine rows were aligned in northeast-southwest orientation and trained using a vertical shoot positioning (VSP) system. In addition, spontaneous vegetation was allowed to grow between the rows, serving as cover crops.

**Fig. 6:**
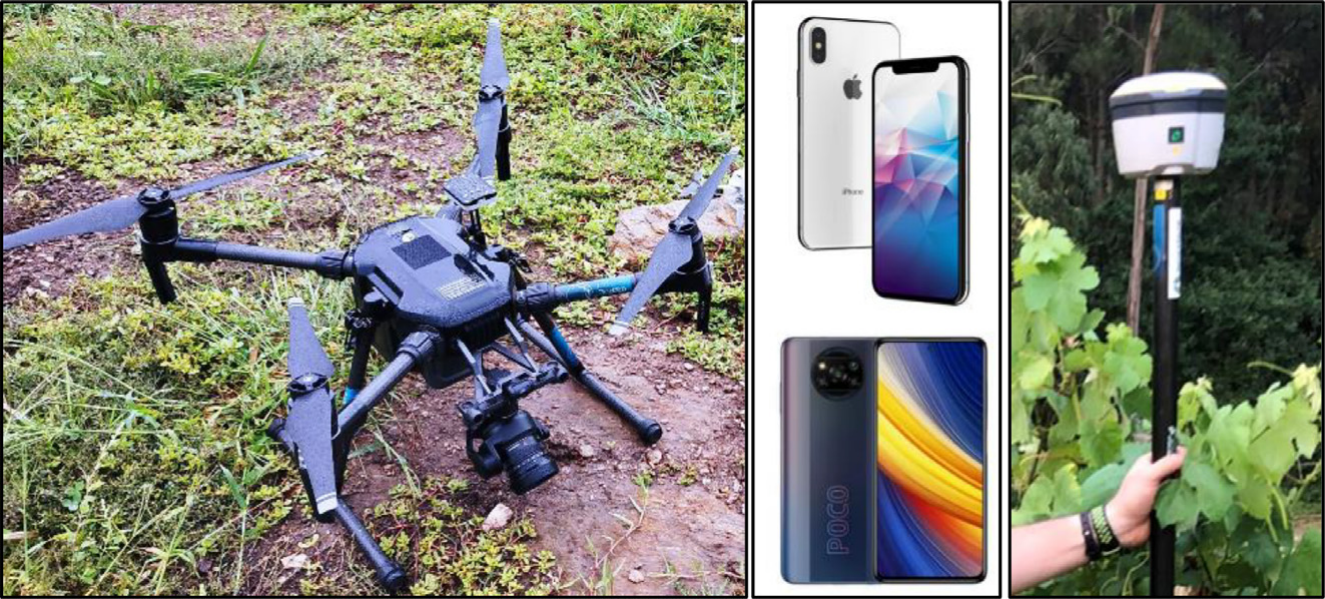
Equipment Used in the Study. Left: DJI M210 Drone. Middle: Smartphones - iPhone X (top) and Xiaomi Poco X3 Pro (bottom). Right: Trimble R2 Integrated GNSS System.

Multispectral images of the vineyards were collected through UAV flights and each plant was documented using smartphones, evaluated for health by professional agronomists, and geotagged using both the GPS of the smartphone and a high-accuracy GNSS RTK system (Fig. 6). Furthermore, aerial images were used to generate point clouds and orthomosaics. This data gathering occurred on the 14th and 15th of July 2022, corresponding to grapevine development stages BBCH77-BBCH79, a period chosen specifically to observe the plants during their vegetative growth phase, before veraison. This timing was strategic for the early detection of diseases and preliminary estimation of yield.

### 4.1 Smartphone images

**Fig. 7:**
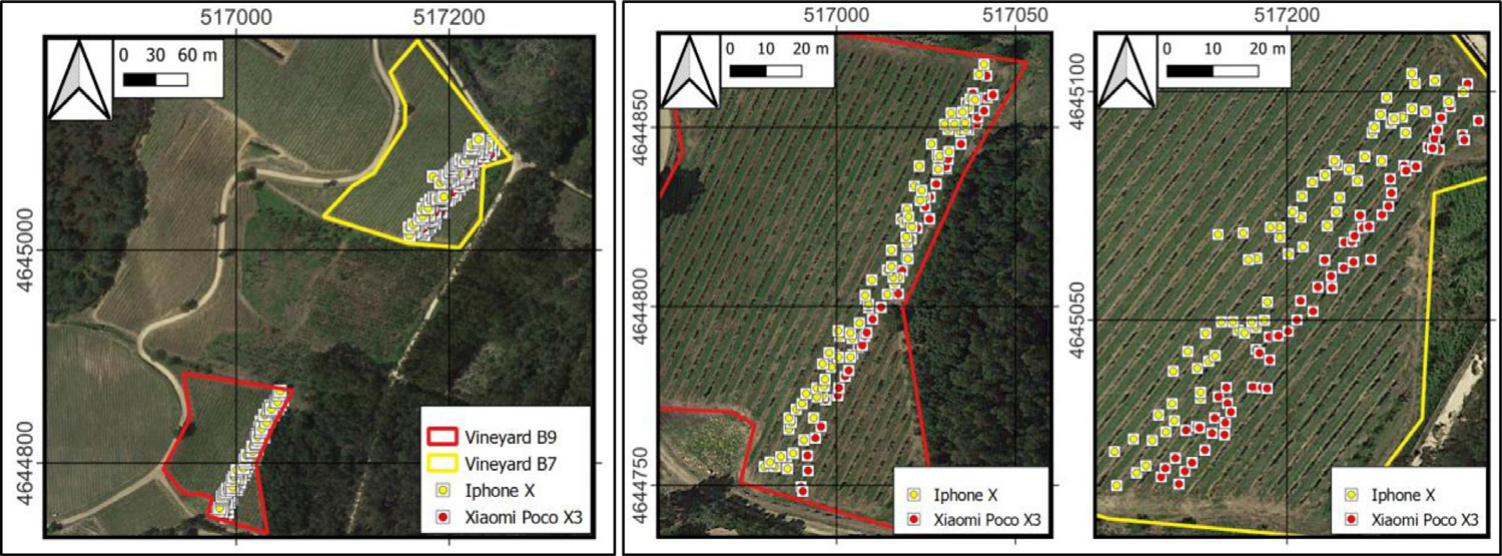
Location of the vineyards. Left: location of the photos in the vineyards. Right: Detail of the photos within vineyard B9 (left) and vineyard B7 (right). ETRS89 / UTM zone 29N, EPSG:25829.

A collection of 253 grape cluster photographs (Fig. 7) were taken across two distinct vineyards during different times of the day (midday and afternoon), utilizing varying light conditions. These images were captured with two smartphones: the Xiaomi Poco X3 Pro (Xiaomi Corporation, Beijing, China) and the iPhone X (Apple Inc., Cupertino, USA). These devices are indicative of typical consumer-grade smartphones and were selected for their relevance to the daily gadgets employed by farmers. The Xiaomi Poco X3 Pro is equipped with a 48 MP main camera and a 6.67 inch screen, and runs on a Snapdragon 860 processor. In contrast, the iPhone X features a 12 MP main camera and operates on the Apple A11 Bionic chipset. The choice of these smartphones was influenced by their distinct operating systems, with Android for the Xiaomi Poco X3 Pro and iOS for the iPhone X, representing the two predominant platforms in the mobile market. This selection underscores the broad accessibility and application of contemporary smartphone technology among farmers, who are progressively embracing digital instruments for farming activities.

### 4.2 UAV flights and multispectral sensor

The aerial surveys were carried out with an Unmanned Aerial System (UAS) that included a UAV DJI Matrice 210 V2 RTK multi-rotor platform (developed by DJI Sciences and Technologies Ltd., Shenzhen, Guangdong, China), equipped with a Micasense Altum sensor (from AgEagle Sensor Systems Inc., Wichita, Kansas, USA). The DJI Matrice 210 V2 RTK is an advanced drone offering outstanding features for professional applications. Weighing approximately 4.91 kg when equipped with two TB55 batteries, this drone is capable of flights of up to 34 min without a charge and 24 min with a take-off weight of 6.14 kg. Its payload capacity is 1.34 kg for the 210 V2 model and 1.23 kg for the 210 RTK V2 version, allowing it to carry a variety of sensors and accessories. In terms of navigation and stability, it offers a hovering accuracy of ± 0.5 m vertical and ± 1.5 m horizontal with GPS, improving to ± 0.1 m with the downward vision system activated or when using the RTK model. It can reach top speeds of up to 81 km/h in S and A modes, and up to 61.2 km/h in P mode while maintaining wind resistance of up to 12 m/s. Additionally, it is designed to operate in a temperature range of –20 ° to 50 °C and has an IP43 ingress protection rating, indicating its resistance to particles and water, making it suitable for use in harsh environmental conditions.

**Fig. 8:**
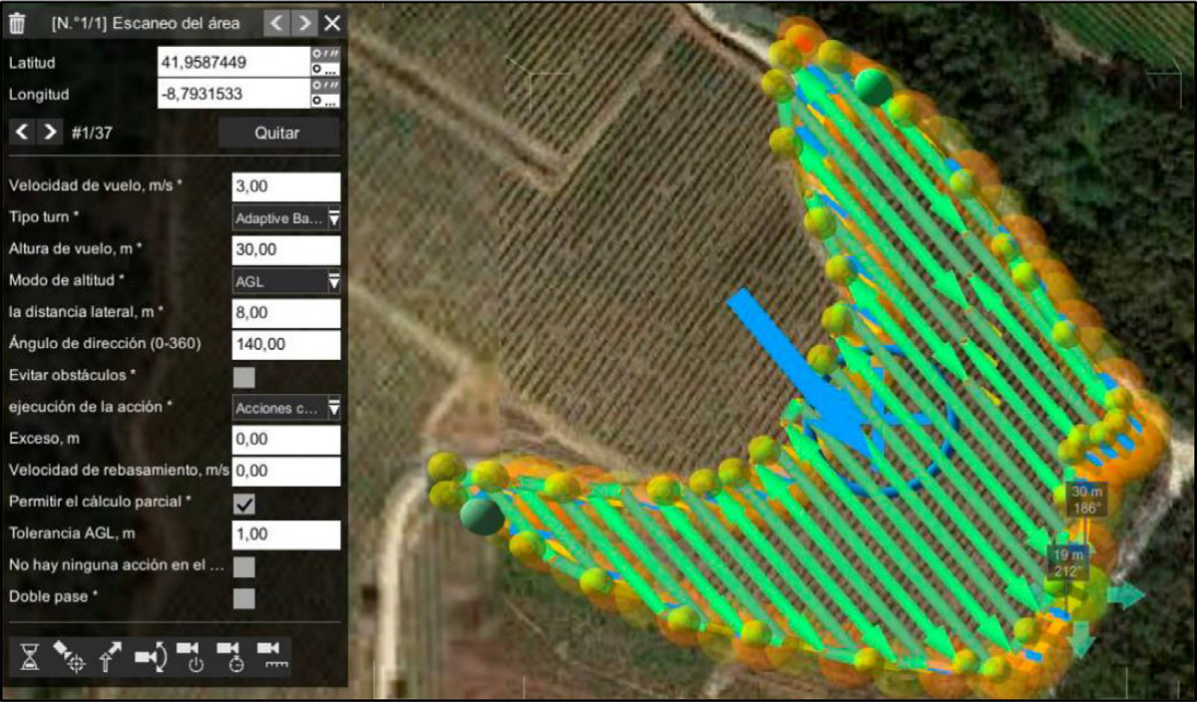
UAV flight survey path for multispectral images at 30m height and nadir angle.

For each mission, the designated flying altitude was set at 30 m above ground level (AGL), with a cruising speed of 3 meters per second, positioning the sensor directly downward (nadir angle) and ensuring both side and frontal overlap of 80 %. The flight routes were planned and executed along the vineyard rows, utilizing a combination of UgCS (SPH Engineering, Riga, Latvia) and DJI PILOT 2 software for flight planning and operational control, respectively (Fig. 8).

The Micasense ALTUM camera sensor captures imagery across six distinct spectral bands, blue, green, red, near-infrared, red edge, and thermal, which are stored as separate, geotagged TIF files for each capture event. Table 2 shows the key specifications of the sensor, according to the information provided by the manufacturer [18].

**Table 2:** Key Specifications for the Micasense ALTUM.

	Multispectral	Thermal
Pixel size	3.45 µm	12 µm
*Resolution*	2064 × 1544 px (3.2 MP × 5 imagers)	160 × 120 px (0.01 K)
*Aspect ratio*	04:03	04:03
*Sensor size*	7.12 × 5.33 mm (8.9 mm diagonal)	1.92 × 1.44 mm
*Focal length*	8 mm	1.77 mm
*Field of view (h x v)*	48° × 36.8°	57° x 44.3°
*Thermal sensitivity*	n/a	< 50 mK
*Thermal accuracy*	n/a	+/- 5 K
*Output bit depth*	12-bit	14-bit
*GSD @ 120 m (∼400 ft)*	5.2 cm	81 cm
*GSD @ 60 m (∼200 ft)*	2.6 cm	41 cm
*Imager/band number*	Blue/1; Green/2; Red/3; NIR/4; Red edge/5	Thermal/6
*Band/Center±Bandwidth*	Blue/475 nm ± 20 m; Green/560 nm ± 20 nm; Red/668 nm ± 10 nm; Red edge/717 nm ± 10 nm; Near infrared/840 nm± 40 nm	Thermal/11 µm ± 6 µm

To produce the 3D point clouds and orthomosaics, the processing started with the organization and verification in PIX4D of multispectral images captured by the Altum sensors, ensuring accurate georeferencing and spectral calibration. Calibration is conducted using images from the Micasense calibrated reflectance panel taken after each flight. Upon importing these images into PIX4D, the project is configured by fine-tuning the specific settings of the Altum sensor. The preliminary alignment of images identifies matching points essential for the 3D reconstruction process. Following this step, a detailed dense point cloud for each spectral band is generated, meticulously mapping the physical landscape of the target area. The next steps involve classifying ground points and creating Digital Elevation Models (DEMs), which serve as a basis for crafting multispectral orthomosaics through geometric image correction, thus ensuring a precise and consistent depiction of the terrain. The final phase involves the analysis, validation, and exportation of the finished point clouds and orthomosaics.

### 4.3 Geospatial data acquisition

The accurate locations of the trunks were taken using a Trimble R2 Integrated GNSS system with a TSC3 Controller (Trimble Inc., California, USA). Trimble R2 is a GNSS receiver designed for high-accuracy data collection in GIS and surveying applications. The device offers a versatile setup, supporting multiple global satellite constellation systems, including GPS, GLONASS, Galileo, and BeiDou. It delivers real-time positioning accuracy from sub-meter to centimetre level, catering to a broad spectrum of geospatial tasks. The receiver is compatible with a range of mobile devices via Bluetooth or USB, enabling integration with existing technology platforms. Notably, the Trimble R2 meets military specifications MIL-STD-810G for ruggedness and holds an IP65 rating for dust and water resistance. Its compact form factor and one-button operation facilitate ease of use in the field, enhancing productivity in diverse environmental conditions.

## Limitations

Although the "EscaYard" dataset could be highly valuable for research in precision viticulture and disease detection, some limitations should be noted. First, using only certain smartphones for data collection might lead to bias because the quality of data depends on the cameras of these phones, and differences in camera quality can affect how uniform and comparable the data are. Also, the dataset mainly covers vineyards in Tomiño, Pontevedra, Galicia, which means the findings might not apply to other areas or different types of vines. Lastly, the use of specific software to create the point clouds and orthomosaics might introduce another source of bias.

## Ethics Statement

The present work meets the ethical requirements for publication in Data in Brief. The work does not involve human subjects, animal experiments, or any data collected from social media platforms.

## Declaration of Generative AI and AI-assisted Technologies in the Writing Process

During the preparation of this work, the authors used ChatGPT in order to improve the English language and avoid orthographic errors. After using this tool/service, the authors reviewed and edited the content as needed and take full responsibility for the content of the publication.

## CRediT authorship contribution statement

**Sergio Vélez:** Investigation, Visualization, Methodology, Data curation, Writing – original draft. **Mar Ariza-Sentís:** Visualization, Writing – review & editing, Methodology, Data curation. **João Valente:** Conceptualization, Supervision, Writing – review & editing.

## Data Availability

Precision viticulture dataset for detailed vineyard mapping composed of geotagged smartph-one ground images, phytosanitary status, UAV orthomosaics, 3D point clouds, and RTK GNSS data-Northern Spain, J (Original data) (Zenodo).
